# Grit Dimensions, Obsessive–Compulsive Symptomatology, and Psychological Distress: A Cross-Sectional Comparison of Two Directional Mediation Models

**DOI:** 10.3390/brainsci16101006

**Published:** 2026-09-23

**Authors:** Kurt Leroy Hoffman, Gilberto Manuel Galindo-Aldana, Esmeralda García-Torres, Hugo Cano-Ramírez

**Affiliations:** 1 Animal Reproduction Research Center, Autonomous University of Tlaxcala, Tlaxcala 90120, Mexico; rexvitro@hotmail.com; 2 Laboratory of Neuroscience and Cognition, Facultad de Ciencias Administrativas, Sociales e Ingeniería, Universidad Autonoma de Baja California, Mexicali 21700, Mexico; 3 Center for Research in Genetics and Environment, Autonomous University of Tlaxcala, Tlaxcala 90120, Mexico

**Keywords:** Grit-O, obsessive–compulsive symptoms, psychological distress, mediation model

## Abstract

**Highlights:**

**What are the main findings?**

- Consistency of Interest (CI) and Perseverance of Effort (PE)—were associated with fewer OC symptoms in undergraduate students, while OC symptoms were positively associated with psychological distress.
- Overall, CI and PE were negatively associated with OC symptoms and psychological distress in both the forward and reverse directional path models. However, PE and CI may have direct negative associations with anxiety and depression, respectively, while OC symptoms may partially mediate the associations between PE and anxiety and between PE and depression.

**What are the implications of the main findings?**

- Both directional models present the same associative structure: Grit subscales were negatively associated with OC symptomatology, and OC symptomatology was positively associated with distress.
- In university students, psychoeducational interventions that increase PE and CI may confer resilience to anxiety and depression, respectively. Moreover, psychological interventions that target subclinical OC symptoms may further strengthen resilience to psychological distress.

**Abstract:**

**Background**: Mental health research has been increasingly oriented toward understanding how protective factors buffer psychological distress and support emotional stability, but the mechanisms underlying goal-oriented behavior remain less studied. One of those mechanisms is Grit, defined as a concept with two dimensions: Consistency of Interest (CI) and Perseverance of Effort (PE). Although these factors may share behavioral components with obsessive–compulsive (OC) symptomatology, the specific statistical contribution of OC symptoms to the association of Grit with psychological distress has not been modeled. **Methods**: An exploratory cross-sectional study was conducted with 333 undergraduate students from a Mexican University. Participants completed a battery of validated Spanish-language instruments: Grit-O (CI and PE subscales), OCI-R, and DASS-21 (depression, anxiety, and stress subscales). **Results**: Overall, Grit-O subscales were negatively associated with both OCI-R and DASS-21 subscales, while OCI-R and DASS-21 subscale scores were positively correlated. Six forward mediation models were estimated, examining the indirect effects of CI and PE on depression, anxiety, and stress through OC symptoms. All direct effects of CI and PE on the DASS-21 subscales were statistically significant, with the exception of the CI to depression and PE to anxiety paths. All indirect effects of CI and PE on the DASS-21 subscales through OC symptoms were statistically significant. Six reverse directional sensitivity models revealed statistically significant effects for all direct paths except anxiety to PE and depression to CI. All indirect effects were statistically significant except for the indirect effects of depression and anxiety on PE through OC symptoms. **Conclusions**: These results indicate that CI and PE are negatively associated with OC symptoms and psychological distress in both the forward and reverse directional models. Path models suggest that PE and CI may have direct negative associations with anxiety and depression, respectively, while OC symptoms may partially mediate the associations between PE and anxiety and between PE and depression.

## 1. Introduction

Mental well-being among university students as a determinant for persistence to achieve objectives has typically gone overlooked relative to academic effort and goal-oriented behavior. Research in the field of psychology has identified personality traits that predict success and academic performance, but under stressful situations, the balance between psychological stability and impaired academic function is thin and not usually recognized [1,2].

Duckworth and colleagues [3] proposed the psychological construct of “Grit” to represent the personal characteristic of perseverance and passion to achieve long-term goals. These investigators developed and validated the Grit-O scale, which is a 12-item self-report organized in two subscales: Consistency of Interest (CI), which refers to the individual’s propensity to maintain a similar set of interests over a long period, and Perseverance of Effort (PE), which involves the degree to which people make a lasting effort to meet challenges [4,5]. Grit scores explained a significant proportion of variance in measures of academic success and were unrelated to intelligence quotient (IQ). Although the psychological construct of Grit is closely related to the “Big Five” personality trait conscientiousness, the PE subscale explains variance in academic performance even after controlling for conscientiousness [6,7].

A positive relationship has been reported between academic perseverance and emotional regulation in university students [8,9,10,11,12]. Emotional regulation is the ability to control and manage emotions, thereby facilitating adaptive responses to challenging situations, and it is a necessary skill for coping with academic stress. University students face challenges that impact their psychological well-being, and adaptive strategies of emotional regulation positively impact their ability to maintain motivation and optimal academic performance. While both Grit-CI and Grit-PE subscales were positively associated with reappraisal (an adaptive emotion regulation strategy), expressive suppression (a maladaptive strategy) was negatively associated with CI and positively associated with PE [13]. In first-year medical residents, higher scores on the PE subscale (but not the CI subscale) predicted lower depressive symptoms after one year, even after adjusting for baseline depression [14].

Perseverance is a psychological construct associated with goal-oriented behavior and is characterized by persistence, strength, and tenacity. Perseverance has been associated with subjective well-being, which allows the development of resilience in the face of adversity and failure [15,16,17,18]. Adverse childhood experiences have been related to lower scores on the PE scale, which in turn were linked to increased anxiety and depression [19], suggesting that higher PE could reflect more resilience to psychological distress. Individuals with high resilience report lower depression, anxiety, stress, and obsessive–compulsive (OC) symptoms [20]. Likewise, the personality trait of conscientiousness has been negatively associated with clinical depression [21].

Obsessive–compulsive symptomatology includes obsessions (intrusive, repetitive thoughts or mental images that are difficult to control, lack purpose, and generate distress) and compulsions (repetitive actions or mental events to relieve the distress caused by obsessions) [21]. Several factors are associated with OC symptom severity, including resilience, maladaptive coping strategies, avoidance behaviors, and trauma-related symptoms [22,23,24,25]. Information on the relationship between Grit and obsessive–compulsive symptoms is sparse, and it is limited to a single study that reported a negative association between Grit and severity of hoarding symptoms [26]. Nevertheless, a number of studies have examined the relationship between Conscientiousness and obsessive–compulsive symptoms, finding that extreme expressions of this trait overlap with obsessive–compulsive characteristics, which in turn are associated with depression and decreased well-being [27,28]. Thus, although Grit may confer resilience to depressive symptoms, and extreme conscientiousness (a construct closely related to Grit) is positively associated with obsessive–compulsive traits and depressive symptoms, the possibility that Grit may be positively associated with OC symptoms, and that OC symptoms may partially mediate effects of Grit on depression, has not been explored.

The present study begins to address this gap by exploring the associations between Grit-O subscales (CI and PE), OC symptoms (OCI-R), and psychological distress (depression, anxiety, and stress; DASS-21). We had three objectives: (1) To examine the psychometric characteristics of the items of the Grit-O subscales (CI and PE) in the present sample of university students; (2) to characterize the statistical relationships between Grit, OC symptoms, depression, anxiety, and stress; and (3) to construct and evaluate path models that assess whether OC symptoms mediate the relationship between Grit and depression, anxiety, or stress Although this study was exploratory, we predicted that Grit would be inversely related to psychological distress, while being positively associated with OC symptoms, and that OC symptoms would negatively mediate the effects of Grit on psychological distress.

## 2. Materials and Methods

This research is a cross-sectional, exploratory, and descriptive study. Non-probability convenience sampling was used, as the sample depended exclusively on individuals’ willingness to participate in the study. Students were approached in their classrooms where the study was described, and participation was entirely voluntary. The selection criteria were enrollment as a student at the institution, voluntary agreement to participate, provision of informed consent, and completion of the survey. No exclusion criteria were defined. The sample consisted of 333 undergraduate students from a Mexican university with a mean age of 19.15 years (SD = 2.36; range: 17–34 years). A total of 242 participants identified as women (72.7%) and 91 as men (27.3%).

### 2.1. Procedure and Ethical Considerations

Data were collected in 2023 through an online battery administered via Google Forms configured to allow only one response per participant, with a battery of standardized psychological tests in Spanish including the Grit-O scale [29], OCI-R [30,31], and DASS-21 [32,33]. No email addresses or IP addresses were collected from participants. In accordance with Article 23 of the Regulations of the Mexican General Health Law on Health Research [34], studies based on anonymous surveys or questionnaires that involve no physical, psychological, or social intervention are classified as no-risk research; written informed consent from parents or legal guardians of minor participants was, therefore, not required. Confidentiality was maintained throughout data collection, storage, and analysis. No missing data were presented for the variables of the study; the sample (*n* = 333) included complete cases as a condition of the application, and no case deletion procedures were necessary. The study followed international Helsinki and regional ethical regulations and was approved by the Research Ethics Committee of the Faculty of Odontology, Universidad Autónoma de Tlaxcala (approval number 156/2023).

### 2.2. Measures

#### 2.2.1. Grit

Grit was evaluated with the Original Grit Scale in Spanish (Grit-O) [29], a self-reported scale of 12 items with two subscales of 6 items (Consistency of Interest and Perseverance of Effort), and a Likert 5-point scale from 1 to 5. CI items are negatively worded and need to be reverse-scored before the analysis. Higher scores of the scales mean greater CI and PE.

#### 2.2.2. Obsessive–Compulsive Symptomatology

Obsessive–compulsive symptomatology was evaluated with the Obsessive–Compulsive Inventory-Revised (OCI-R) [30] with the Spanish adaptation by Fullana et al. (2005) [31]. This inventory is an 18-item self-report with six subscales with 3 items each: obsessing, washing, checking, ordering, hoarding, and neutralizing. The scale includes a 5-point Likert scale ranging from 0 to 4. The total score is computed as the sum of all the items; a higher score indicates greater severity. A total score of 21 is considered the cutoff to distinguish between clinical and non-clinical populations.

#### 2.2.3. Psychological Distress

Psychological distress was evaluated with the 21-item version of the depression, anxiety, and stress (DASS-21) Spanish version [33]. This scale comprises 3 subscales of 7 items rated with a 4-point item scale from 0 to 3. The subscale scores were doubled and computed as the sum of the seven items for comparisons. Severity cutoffs were considered non-normative but exploratory (depression ≥ 14, anxiety ≥ 10, stress ≥ 19) in the study and do not constitute diagnostic categories.

### 2.3. Data Analysis

Descriptive statistics, reliability estimates, and confirmatory factor analysis were performed using JASP version 0.96 for Windows [35], and confirmatory factor analysis and mediation analyses were conducted in jamovi version 2.6 [36] using the jamovi Advanced Mediation Models (jAMM) module [37,38,39]. Indirect effects were estimated with non-parametric bootstrapping (B = 5000 resamples) and bias-corrected 95% confidence intervals.

Normality was assessed using the Shapiro–Wilk test for males (*n* = 91) and females (*n* = 242). The Shapiro–Wilk test included significant departures from normality for OCI-R total and the subscales of DASS-21 (*p* < 0.001; skewness range 0.41–0.76; Supplementary Table S1), and the Grit-CI subscale and Grit-O total did not include deviations from normality. To observe the influence of common method bias, a single-factor test was conducted with all the scale items in an unrotated exploratory factor analysis constrained to a single factor. The extracted factor accounted for 32.9% of the total variance.

Due to the non-normality in OCI-R and DASS-21 scores, Spearman correlations were calculated for the variables as a robustness verification. Coefficients were comparable across methods. Pearson’s r for asymptomatically sampling distributions was retained for the correlation matrix for consistency between the descriptive and inferential results. However, Spearman’s rho was calculated along with Pearson’s r as a sensitivity calculation showing that both converged, indicating correlational findings (Supplementary Table S2). Threshold group comparisons employed a non-parametric Mann–Whitney U test that did not assume normality within subgroups and was adequate for unequal group sizes (Supplementary Table S3).

Mann–Whitney U tests were used for sex-based comparisons. An a priori power analysis was conducted using G*Power 3.1 [40] for the predictor to mediator path (CI and PE with OCI-R total; 2 predictors); a minimum of 107 participants was required to detect a medium effect (f^2^ = 0.15) with α = 0.05 and power = 0.95. The final sample of N = 333 met this requirement. Further sensitivity analyses were conducted with N = 333, α = 0.05, and a single tested predictor within a model containing three predictors; the design was sensitive to effects of f^2^ ≥ 0.024 at power = 0.80 and f^2^ ≥ 0.039 at power = 0.95 (ΔR^2^ = 0.023 and 0.038, corresponding to partial correlations of 0.152 and 0.194).

## 3. Results

### 3.1. Two-Factor Model of Grit Structure, Descriptive Statistical Analysis, and Internal Consistency of the Grit-O Scale

The Grit-O total score (M = 3.32, SD = 0.58, range = 1.58–4.92) captured the contribution of both subscales. CI (M = 2.97, SD = 0.80, range: 1.00–5.00) scored lower than PE (M = 3.66, SD = 0.71, range: 1.50–5.00). Also, Grit-O (Table 1) demonstrated acceptable internal consistency at the total scale scores (McDonald’s ω = 0.710), CI (McDonald’s ω = 0.76), and PE (McDonald’s ω = 0.76). Omega coefficients for the OCIR and DASS21 scales of this same university student sample have been published in a previous study [41].

A confirmatory factor analysis (CFA) was conducted to evaluate the original factor structure of the Grit-O scale. The model showed moderate fit, χ^2^ (53) = 151.30 and *p* < 0.001; CFI = 0.893; TLI = 0.866; RMSEA = 0.075 [90% CI: 0.0061–0.089]; and SRMR = 0.082. Factor loadings (Table 2) indicate that most items loaded satisfactorily within factors. However, some of the items fell below the conventional 0.40 threshold, with Consistency of Interest at G11 (0.37) and Perseverance of Effort at G4 (0.29).

Given the unbalanced sex distribution, which is due primarily to the characteristics of the student population and not a selection bias [42], preliminary Mann–Whitney U tests were conducted in an exploratory manner to determine whether Consistency of Interest, Perseverance of Effort, or Grit total scores differed by sex. We found no significant sex-associated differences for CI (U = 9939.000, *p* = 0.170), PE (U = 12040.000, *p* = 0.189), or Grit total (U = 10850.000, *p* = 0.837). Therefore, we did not include gender as a moderating factor in the mediation models.

In order to observe if the Grit-O two-factor structure and items were equivalent across sex, a measurement of invariance was conducted. CI and PE metric invariance were supported, but scalar invariance was not (Table 3). The chi-square difference test for the metric model was non-significant; *p* = 0.182. Fit indices for the models are presented (Table 3); the configural model shows marginal fit, with χ^2^ (106) = 219.776 and *p* < 0.001; CFI = 0.881; TLI = 0.852; RMSEA = 0.080 [90% CI: 0.065, 0.095]; and SRMR = 0.091. Metric invariance for gender comparison of association among variables was supported, producing a meaningful decrement, with ΔCFI = −0.004, ΔRMSEA = −0.002, Δχ^2^(10) = 13.802, and *p* = 0.182. However, scalar invariance was not supported, with ΔCFI = −0.040, Δχ^2^(10) = 48.041, and *p* < 0.001. ΔRMSEA remained within the recommended threshold (+0.008).

Pearson correlations were used to examine associations between the OCI-R symptoms and Grit factors (Table 4); all OCI-R subscales were negatively associated with Grit scores (CI, PE, and total), indicating that higher Grit corresponded to lower OC symptom severity. CI presented stronger associations with OCI-R subscales than PE; specifically, the strongest correlations were observed between Grit total and OCI-R total (r = −0.447), followed by obsessing (r = −0.410) and neutralizing (r = −0.403).

### 3.2. Exploratory Analysis of Psychological Distress, Obsessive–Compulsive Symptomatology, and Grit Factors

For descriptive purposes, Grit-O scores (CI, PE, and total) were compared between participant subgroupings of high and low scores on the DASS-21 subscales; the respective severity cutoff points are depression ≥ 14, anxiety ≥ 10, and stress ≥ 19 (Supplementary Table S3). Mann–Whitney U tests were conducted for each Grit variable (CI, PE, total) across all three DASS-21 subscale groupings. The same analysis was performed for high and low subgroupings of OCI-R scores (severity cutoff is >21 points; 29).

For depression, the above threshold group scored lower on Consistency of Interest (U = 19,160, *p* < 0.001, r = −0.390), Perseverance of Effort (U = 17,840, *p* < 0.001, r = −0.294), and Grit total (U = 20,070, *p* < 0.001, r = –0.456). For anxiety, similar results were found for Consistency of Interest (U = 18,210, *p* < 0.001, r = −0.384), Perseverance of Effort (U = 16,130, *p* < 0.001, r = −0.226), and Grit total (U = 18,370, *p* < 0.001, r = −0.396), with high symptom scores associated with lower Grit scores.

Likewise, for stress, the high-scoring subgroup presented significantly lower scores for Consistency of Interest (U = 18,960, *p* < 0.001, r = −0.403), Perseverance of Effort (U = 17,040, *p* < 0.001, r = −0.261), and Grit total (U = 19,320, *p* < 0.001, r = −0.430). Regarding OCI-R (*n* = 169, 164), Consistency of Interest was lower in the high OCI-R scoring subgroup (M = 2.732, SD = 0.760) than the low scoring subgroup (M = 3.211, SD = 0.775), with U = 8978, *p* < 0.001, and r = −0.352; the same pattern was presented for Perseverance of Effort (U = 11,565, *p* = 0.009, r = −0.166) and Grit total (U = 9095, *p* < 0.001, r = −0.344).

Furthermore, to examine the relationship of each Grit-O subscale to OCI-R and DASS-21 scores, partial correlations were employed controlling for the subscale. Controlling for PE, CI remained significantly and negatively associated with OCI-R total scores, depression, anxiety, and stress (Table 5). Similarly, controlling for CI, PE retained significant negative associations with OCI-R total, depression, anxiety, and stress.

### 3.3. Indirect Effects Analysis of Grit Subscales on Psychological Distress via OCI-R

To examine if obsessive–compulsive symptoms statistically account for the association between Grit and psychological distress, bootstrap mediation analyses were conducted for each Grit subscale (CI and PE) and DASS-21 subscales, obtaining twelve models with OCI-R total score as the mediator. In each model, three paths were estimated: the a-path represents the association between the Grit-O subscales and OC symptomatology, the b-path represents the association between OC symptomatology and psychological distress, and the c’ path is the association between the Grit-O subscale and psychological distress after adjusting for OC symptomatology.

As a complementary analysis, two adjusted a-paths were compared as separate simultaneous two model predictors with the difference in unstandardized a-path coefficients tested by reparametrizing the simultaneous two-predictor model using the following formulas: sum = CI + PE and difference = CI − PE. These paths were entered as predictors of OCI-R total in place of CI and PE; the two adjusted a-paths did not differ significantly, with B(difference) = −2.284, 95% CI [−5.256, 0.688], t (330) = −1.512, and *p* = 0.132. Tolerance = 0.983 and VIF = 1.017 for sum and difference indicate no multicollinearity concern.

It is noted that in a two-predictor simultaneous model (CI and PE as predictors of OC symptoms), the b-path (OC symptoms with psychological distress) is shared across predictors; each outcome model is reported once (Table 6). The direct path (c’) represents the association between the Grit-O subscales and psychological distress after adjusting for OC symptomatology. The indirect path (ab) quantifies the extent to which OC symptomatology statistically accounts for that association. CI and PE were each inversely associated with OC symptomatology (CI: B = −6.807, SE = 0.921, *p* < 0.001; PE: B = −4.523, SE = 1.047, *p* < 0.001). OC symptomatology was positively associated with all three distress outcomes: depression (B = 0.414, SE = 0.033, *p* < 0.001), anxiety (B = 0.408, SE = 0.031, *p* < 0.001), and stress (B = 0.428, SE = 0.029, *p* < 0.001). The direct paths (c) of PE with depression and stress were negative and statistically significant (B = −4.719, SE = 0.767, *p* < 0.001; B = −3.417, SE = 0.718, *p* < 0.001, respectively), as were direct paths of CI with anxiety and stress (B = −4.690, SE = 0.637, *p* < 0.001; B = −4.586, SE = 0.631, *p* < 0.001, respectively). Direct paths from PE to anxiety and CI to depression were not statistically significant. All indirect paths through OC symptoms were negative and statistically significant.

In the reverse specification (Panel B), where the Grit subscales were presented as outcomes, each distress subscale was positively associated with OC symptomatology (depression: B = 0.889, SE = 0.057; anxiety: B = 0.980, SE = 0.059; stress: B = 1.024, SE = 0.057; all *p* < 0.001). OC symptomatology was, in turn, inversely associated with CI in three models (depression: B = −0.017, SE = 0.004, *p* < 0.001; anxiety: B = −0.013, SE = 0.004, *p* < 0.001; stress: B = −0.013, SE = 0.004, *p* < 0.001), whereas the association with PE was statistically significant only in the anxiety model (B = −0.010, SE = 0.003, *p* = 0.003; depression: B = −0.004, SE = 0.003, *p* = 0.238; stress: B = −0.006, SE = 0.003, *p* = 0.070). The direct paths of depression and stress to PE were negative and statistically significant, as were the direct paths of anxiety and stress to CI. By contrast, direct paths of anxiety with PE and depression with CI were not statistically significant. All indirect paths through OC symptoms were negative and significant, with the exception of depression to PE and stress to PE. Taken together, the results of forward and reverse specification models suggest direct negative path associations from CI to depression and from PE to anxiety, as well as indirect negative path associations from PE to stress and from PE to depression through OC symptoms.

## 4. Discussion

The present study aimed to contribute to the literature that has examined associations among Grit, obsessive–compulsive symptomatology, and psychological distress, given that these associations may be shaped by unclear interactions arising from sociocultural differences [43,44]. Contrary to our prediction, we found that higher CI and PE were associated with lower OC symptoms. As expected, higher OC symptoms were related to greater psychological distress for the three DASS-21 subscales, and both Grit subscales were negatively correlated with psychological distress. Path analysis revealed bidirectional associations between these variables, direct negative associations of CI with depression and of PE with anxiety, and indirect negative associations of PE with stress and depression through OC symptoms.

With regard to the first study objective, we determined that the Grit-O scale demonstrated acceptable internal consistency at the levels of total and subscale scores in the present sample of university students. The two-factor structure of the Grit-O scale was supported by our data, with most items showing strong factor loadings on Consistency of Interest (CI) and Perseverance of Effort (PE). CI and PE were only moderately correlated.

A second objective was to explore the relationship between Grit-O scores (CI, PE, and total scores) and psychological distress or OC symptoms. We found that, overall, higher Grit-O scores were associated with lower OC, depression, anxiety, and stress symptoms. This relationship was observed both when considering categorical subgroupings of high and low symptom presentation (based on established severity cutoff points), as well as in correlational analyses. Thus, the subgroups of participants with high scores on the OCI-R and DASS-21 subscales (depression, anxiety, and stress) consistently showed significantly lower Grit-O total and subscale scores compared with subgroups that presented low OCI-R and DASS-21 scores. Correlational analyses further confirmed the negative association between Grit-O scores and OC, depression, anxiety, and stress symptoms.

Notably, the statistical effect of high and low symptom subgroupings was stronger for CI and for Grit-O total than for PE, indicating that the CI subscale may be more sensitive to psychological distress and OC symptoms than the PE subscale. Furthermore, partial correlation analysis suggested that CI may be more strongly associated with OC symptoms, anxiety, and stress, while PE may be more strongly associated with depression. The observation that CI and PE differ with respect to their relationships with psychological distress and OC symptoms may indicate that CI and PE are differentially associated with these symptoms, or conversely, that CI and PE confer differential resilience to these symptoms. Similar negative relationships between Grit and depression, anxiety, stress and OC symptoms have been reported [45,46,47,48].

Some studies have reported positive associations between the Big Five personality trait of conscientiousness and obsessive–compulsive personality disorder [27,28] and obsessive–compulsive disorder [22,49,50]. These positive associations may be due to overlap of the upper range of the conscientiousness scale with the obsessive–compulsive characteristics of perfectionism, fastidiousness, punctiliousness, workaholism, doggedness, and ruminative deliberation [27,28]. Given the close positive association between Grit and conscientiousness, we explored the possibility that Grit might also be positively associated with OC symptoms. Contrary to our prediction, Grit was negatively correlated with OC symptom subscales, and these negative associations were also apparent in the forward (Grit–OC symptoms–psychological distress) and reverse (psychological distress–OC symptoms–Grit) path models. This difference may be due to the absence of maladaptive aspects of extreme Grit (unlike extreme conscientiousness), Grit-O failing to capture such aspects, or the absence of individuals with extreme Grit in our sample.

With respect to our third objective, path analysis revealed statistically significant forward and reverse path associations between Grit, OC symptoms, and psychological distress, consistent with bidirectional relationships among these variables [51,52]. However, when taken together, forward and reverse specification models were also consistent with direct negative associations of CI with depression and of PE with anxiety, as well as indirect negative associations of PE with stress and depression, through OC symptoms. These latter findings are consistent with recent studies showing that higher scores on the PE subscale predicted lower depressive symptoms after one year of medical residency, even after adjusting for baseline depression [14].

## 5. Conclusions

Although mediation analysis cannot establish causal associations, and unmeasured psychological factors may also play a role, the variables captured through the standardized instruments employed here offer relevant information about how the two Grit subscales relate to lower scores on measures of psychological distress. Both CI and PE were inversely associated with OC symptomatology and psychological distress. The present findings suggest that goal-directed perseverance (reflected by the Grit-PE scale) may be associated with lower OC symptomatology and lower psychological distress, and they have significant implications for developing and implementing intervention programs that promote both mental health and academic performance in university students.

In a practical sense, these results could be informative for elaborating and implementing psychological instruments for assessing the relationship between academic success and subclinical psychiatric symptoms, such as psychological distress and OC symptoms, and how the psychological construct of Grit may impact that relationship. Recent studies indicate that Grit can be increased by targeted psychological interventions [53,54], presenting the possibility that early interventions to increase Grit in psychologically vulnerable university students might serve to increase resilience to depression, anxiety, and stress, as well as promote optimal academic performance [55]. Thus, the present findings identify an area of opportunity and provide a foundation for future research and interventions aimed at strengthening Grit, emotional regulation, and resilience.

## 6. Limitations

It is important to emphasize that the observed inverse associations between Grit subscales (CI and PE) scores, OC symptom severity, and psychological distress should not be interpreted as evidence of a causal protective effect of Grit, nor should the mediating role of OC symptoms be taken to imply a unidirectional influence on distress outcomes. The reported mediation analyses constitute statistical decompositions of observed associations rather than tests of causal mechanisms.

The cross-sectional design limits interpretation to a single time point and does not allow conclusions regarding temporal precedence or directionality of effects. Future research employing a longitudinal design would support a more rigorous examination of the temporal ordering of these associations. Furthermore, given the homogeneity of the sample, future research should include students from other universities and same-age peers without a university background, to determine if the findings are generalizable. Finally, replicating this study with more diverse samples could reveal motivational and cognitive profiles associated with Grit and psychological distress that may vary cross-culturally.

## Figures and Tables

**Table 1:** Internal consistency of Grit-O (N = 333). k = number of items; ω = McDonald’s omega; 95% CI.

Scale	*k*	ω	95% CI
Consistency of Interest	6	0.760	[0.720, 0.799]
Perseverance of Effort	6	0.759	[0.719, 0.799]
Grit-O Total	12	0.707	[0.661, 0.753]

**Table 2:** Standardized factor loadings via maximum likelihood (ML) for the two-factor Grit-O model (CI = Consistency of Interest; PE = Perseverance of Effort). Factor correlation: r = 0.31. All factor loadings were statistically significant (*p* < 0.01); loadings for G4 and G11 fell below 0.40 and were retained for discussion purposes.

Items	Factor	Loading
G2. New ideas and projects sometimes distract me from previous ones.	CI	0.600
G3. My interests change from year to year.	CI	0.540
G5. I have been obsessed with a certain idea or project for a short time but later lost interest.	CI	0.760
G7. I often set a goal but later choose to pursue a different one.	CI	0.470
G8. I have difficulty maintaining my focus on projects that take more than a few months to complete.	CI	0.680
G11. I become interested in new pursuits every few months.	CI	0.370
G1. I have overcome setbacks to conquer an important challenge.	PE	0.550
G4. Setbacks don’t discourage me.	PE	0.290
G6. I am a hard worker.	PE	0.620
G9. I finish whatever I begin.	PE	0.680
G10. I have achieved a goal that took years of work.	PE	0.680
G12. I am diligent.	PE	0.720

**Table 3:** Measurement invariance of Grit-O across sex (N = 333; female *n* = 242, male *n* = 91); multi-group confirmatory factor analysis estimated with maximum likelihood (ML). χ^2^ = chi-square; df = degrees of freedom; CFI = comparative fit index; TLI = Tucker–Lewis index; RMSEA = root mean square error of approximation; SRMR = standardized root mean square residual.

Model	*χ* ^2^	*df*	CFI	TLI	RMSEA [90% CI]	SRMR	Δ*χ*^2^ (Δ*df*)	ΔCFI	ΔRMSEA
Configural	219.776	106	0.881	0.852	0.080 [0.065, 0.095]	0.091	—	—	—
Metric	233.578	116	0.877	0.861	0.078 [0.063, 0.092]	0.095	13.802 (10)	−0.004	−0.002
Scalar	281.619	126	0.838	0.830	0.086 [0.073, 0.100]	0.095	48.041 (10)	−0.040	+0.008

**Table 4:** Pearson correlations between OCI-R subscales, Grit-O subscales, and total scores (** *p* < 0.01, *** *p* < 0.001).

Scale	Grit-CI	Grit-PE	Grit-O total
Washing	−0.309 ***	−0.182 ***	−0.326 ***
Obsessing	−0.323 ***	−0.302 ***	−0.410 ***
Ordering	−0.276 ***	−0.162 **	−0.291 ***
Checking	−0.322 ***	−0.246 ***	−0.375 ***
Neutralizing	−0.403 ***	−0.194 ***	−0.400 ***
Hoarding	−0.299 ***	−0.208 ***	−0.335 ***
OCI-R total	−0.401 ***	−0.274 ***	−0.447 ***

**Table 5:** Partial correlations controlling for each Grit-O subscale (CI and PE) for OCI-R total symptomatology and depression, anxiety, and stress, with 95% CIs from percentile bootstrap (B = 1000). r = partial correlation.

**Consistency of Interest**	***r***	***p***	**95% CI**
OCI-R Total	−0.375	<0.001	[−0.477, −0.275]
Depression	−0.294	<0.001	[−0.392, −0.201]
Anxiety	−0.375	<0.001	[−0.463, −0.287]
Stress	−0.370	<0.001	[−0.461, −0.275]
**Perseverance of Effort**	***r***	***p***	**95% CI**
OCI-R Total	−0.230	<0.001	[−0.340, −0.115]
Depression	−0.320	<0.001	[−0.419, −0.201]
Anxiety	−0.191	<0.001	[−0.308, −0.068]
Stress	−0.253	<0.001	[−0.360, −0.137]

**Table 6:** Forward and reverse mediation models linking Grit-O subscales, OC symptomatology, and psychological distress. Panel A. Forward models (Grit-O subscales as predictors, OCI-R as a mediator, psychological distress as an outcome). Panel B. Reverse models (psychological distress as a predictor, OCI-R as a mediator, Grit-O subscale as an outcome). * *p* < 0.05; ** *p* < 0.01; *** *p* < 0.001.

**Predictor**	**Outcome**	**a-Path B (SE)**	**b-Path B (SE)**	**Total Effect (c)**	**Direct Effect (c’)**	**Indirect Effect (ab)**	**95% Boot CI**
Consistency of Interest	Depression	−6.807 (0.921) ***	0.414 (0.033) ***	−3.783 (0.674) ***	−0.963 (0.599)	−2.820 (0.443)	[−3.846, −1.966]
Perseverance of Effort		−4.523 (1.047) ***		−4.719 (0.767) ***	−2.845 (0.648) ***	−1.874 (0.459)	[−2.938, −0.930]
Consistency of Interest	Anxiety	−6.807 (0.921) ***	0.408 (0.031) ***	−4.690 (0.637) ***	−1.911 (0.554) ***	−2.779 (0.430)	[−3.758, −1.941]
Perseverance of Effort		−4.523 (1.047) ***		−2.574 (0.725) ***	−0.728 (0.600)	−1.846 (0.449)	[−2.848, −0.815]
Consistency of Interest	Stress	−6.807 (0.921) ***	0.428 (0.029) ***	−4.586 (0.631) ***	−1.670 (0.530 ) **	−2.915 (0.442)	[−3.902, −2.086]
Perseverance of Effort		−4.523 (1.047) ***		−3.417 (0.718) ***	−1.479 (0.574) *	−1.937 (0.468)	[−2.980, −0.936]
**Predictor**	**Outcome**	**a-path B (SE)**	**b-path B (SE)**	**Total effect (c)**	**Direct effect (c’)**	**Indirect effect (ab)**	**95% Boot CI**
Depression	Consistency of Interest	0.889 (0.057) ***	−0.017 (0.004) ***	−0.024 (0.004) ***	−0.009 (0.005)	−0.015 (0.003)	[−0.022, −0.009]
	Perseverance of Effort		−0.004 (0.003)	−0.023 (0.003) ***	−0.020 (0.004) ***	−0.003 (0.003)	[−0.010, 0.002]
Anxiety	Consistency of Interest	0.980 (0.059) ***	−0.013 (0.004) ***	−0.031 (0.004) ***	−0.018 (0.005) ***	−0.013 (0.004)	[−0.021, −0.005]
	Perseverance of Effort		−0.010 (0.003) **	−0.017 (0.004) ***	−0.007 (0.005)	−0.010 (0.003)	[−0.017, −0.003]
Stress	Consistency of Interest	1.024 (0.057) ***	−0.013 (0.004) ***	−0.031 (0.004) ***	−0.018 (0.005) **	−0.013 (0.004)	[−0.022, −0.005]
	Perseverance of Effort		−0.006 (0.003)	−0.020 (0.004) ***	−0.014 (0.005) **	−0.006 (0.004)	[−0.014, 0.0003]

## Data Availability

Data are not publicly available because participants did not consent to public deposition. De-identified data may be shared upon reasonable request, subject to ethics committee approval.

## Supplementary Materials

The following supporting information can be downloaded at: https://www.mdpi.com/article/10.3390/brainsci16101006/s1, Table S1. Descriptive statistics and tests of univariate normality for the study variables. Table S2. Independent-samples comparisons of study variables by sex and by age. Table S3. Descriptive comparison of GRIT-O scores between DASS-21 and OCI-R severity groups above and below severity bands.
