# Supplementary material for: Grit Dimensions, Obsessive–Compulsive Symptomatology, and Psychological Distress: A Cross-Sectional Comparison of Two Directional Mediation Models
Source: Brain Sci. 2026 Sep 23;16(10):1006. doi: 10.3390/brainsci16101006 (PMC13702161; doi:10.3390/brainsci16101006)
Supplement: Supplementary file 1 [file brainsci-16-01006-s001.zip › brainsci-4529885-supplementary.pdf]

Supplementary Table S1. Descriptive statistics and tests of univariate normality for the study variables (M = mean; SD = standard deviation; W = Shapiro–Wilk statistic).

Variable	*M*	*SD*	Skewness	Kurtosis	*W*	*p*
Consistency of Interest	2.97	0.80	–0.082	–0.254	.992	.063
Perseverance of Effort	3.66	0.71	–0.464	0.074	.978	< .001
Grit total	3.32	0.58	0.074	0.044	.994	.181
OCI-R total	23.06	14.93	0.476	–0.572	.962	< .001
DASS-21 Depression	13.62	10.88	0.760	–0.158	.927	< .001
DASS-21 Anxiety	13.90	10.22	0.681	–0.335	.939	< .001
DASS-21 Stress	17.98	10.26	0.409	–0.565	.968	< .001

Supplementary Table S2. Independent-samples comparisons of study variables by sex and by age (median). Age comparison (≤19 vs. >19 years). • Significant only Mann–Whitney, does not survive correction for multiple comparisons.

	**Sex**		**Age**	
**Variable**	**Welch p**	**Mann–Whitney p**	**Welch p**	**Mann–Whitney p**
Grit-O Total	.945	.837	.690	.761
Consistency of Interest	.203	.170	.225	.175
Perseverance of Effort	.174	.189	.497	.446
DASS-21 Depression	.371	.201	.706	.297
DASS-21 Anxiety	.109	.113	.259	.206
DASS-21 Stress **	.004	.002	.669	.731
OCI-R Washing	.268	.177	.674	.530
OCI-R Obsessing *	.011	.013	.617	.559
OCI-R Ordering *	.010	.013	.918	.621
OCI-R Checking	.532	.362	.520	.408
OCI-R Neutralizing	.288	.219	.489	.423
OCI-R Hoarding •	.072	.116	.119	.048

OCI-R Total	.097	.103	.417	.269

Supplementary Table S3. Descriptive comparison of GRIT-O scores between DASS-21 and OCI-R severity groups above and below severity bands (Depression ≥ 14, Anxiety ≥ 10, Stress ≥ 19; Lovibond & Lovibond, 1995). Effect sizes reported as rank-biserial r. OCI-R total (above, n=169; below=164), depression (above, n = 154; below, n = 179), anxiety (above, n = 204; below, n = 129), Stress (above, n = 140; below, n = 193).

		**Above**		**Below**				
**Grouping Variable**	**Variable**	***M***	***SD***	***M***	***SD***	***U***	***p***	***r***
OCI-R	GRIT-CI	2.732	0.760	3.211	0.775	18,737.50	<.001	–.352
	GRIT-PE	3.551	0.747	3.779	0.643	16,151.00	.009	–.166
	GRIT-O Total	3.142	0.570	3.495	0.528	18,621.00	<.001	–.344
Depression	GRIT-CI	2.687	0.759	3.209	0.762	19,160.00	<.001	–.390
	GRIT-PE	3.464	0.733	3.835	0.636	17,840.00	<.001	–.294
	GRIT-O Total	3.076	0.525	3.522	0.540	20,070.00	<.001	–.456
Anxiety	GRIT-CI	2.769	0.767	3.283	0.759	18,210.00	<.001	–.384
	GRIT-PE	3.552	0.725	3.840	0.640	16,130.00	<.001	–.226
	GRIT-O Total	3.161	0.556	3.561	0.524	18,370.00	<.001	–.396
Stress	GRIT-CI	2.655	0.728	3.195	0.780	18,960.00	<.001	–.403
	GRIT-PE	3.467	0.748	3.807	0.639	17,040.00	<.001	–.261
	GRIT-O Total	3.061	0.544	3.501	0.528	19,320.00	<.001	–.430
